# Molecular characterization of human HSPCs with different cell fates in vivo using single‐cell transcriptome analysis and lentiviral barcoding technology

**DOI:** 10.1002/ctm2.70085

**Published:** 2024-11-13

**Authors:** Junnan Hua, Ke Wang, Yue Chen, Xiaojing Xu, Guoyi Dong, Yue Li, Rui Liu, Yecheng Xiong, Jiabin Ding, Tingting Zhang, Xinru Zeng, Yuxi Li, Haixi Sun, Ying Gu, Sixi Liu, Wenjie Ouyang, Chao Liu

**Affiliations:** ^1^ College of Life Sciences University of Chinese Academy of Sciences Beijing China; ^2^ BGI Shenzhen China; ^3^ School of Biology and Biological Engineering South China University of Technology Guangzhou China; ^4^ BGI Hemogen Therapeutic Shenzhen China; ^5^ Department of Hematology and Oncology Shenzhen Children's Hospital Shenzhen China

**Keywords:** barcoding technology, hematopoietic stem and progenitor cells, lineage tracing, scRNA‐seq

## Abstract

Hematopoietic stem and progenitor cells (HSPCs) possess the potential to produce all types of blood cells throughout their lives. It is well recognized that HSPCs are heterogeneous, which is of great significance for their clinical applications and the treatment of diseases associated with HSPCs. This study presents a novel technology called Single‐Cell transcriptome Analysis and Lentiviral Barcoding (SCALeBa) to investigate the molecular mechanisms underlying the heterogeneity of human HSPCs in vivo. The SCALeBa incorporates a transcribed barcoding library and algorithm to analyze the individual cell fates and their gene expression profiles simultaneously. Our findings using SCALeBa reveal that HSPCs subset with stronger stemness highly expressed *MYL6B*, *ATP2A2*, *MYO19*, *MDN1*, *ING3*, and so on. The high expression of *COA3*, *RIF1*, *RAB14*, and *GOLGA4* may contribute to the pluripotent‐lineage differentiation of HSPCs. Moreover, the roles of the representative genes revealed in this study regarding the stemness of HPSCs were confirmed with biological experiments. HSPCs expressing *MRPL23* and *RBM4* genes may contribute to differentiation bias into myeloid and lymphoid lineage, respectively. In addition, transcription factor (TF) characteristics of lymphoid and myeloid differentiation bias HSPCs subsets were identified and linked to previously identified genes. Furthermore, the stemness, pluripotency, and differentiation‐bias genes identified with SCALeBa were verified in another independent HSPCs dataset. Finally, this study proposes using the SCALeBa‐generated tracking trajectory to improve the accuracy of pseudo‐time analysis results. In summary, our study provides valuable insights for understanding the heterogeneity of human HSPCs in vivo and introduces a novel technology, SCALeBa, which holds promise for broader applications.

**Key points:**

- SCALeBa and its algorithm are developed to study the molecular mechanism underlying human HSPCs identity and function.
- The human HSPCs expressing *MYL6B, MYO19, ATP2A2, MDN1, ING3*, and *PHF20* may have the capability for high stemness.
- The human HSPCs expressing *COA3, RIF1, RAB14*, and *GOLGA4* may have the capability for pluripotent‐lineage differentiation.
- The human HSPCs expressing *MRPL23* and *RBM4* genes may have the capability to differentiate into myeloid and lymphoid lineage respectively in vivo.
- The legitimacy of the identified genes with SCALeBa was validated using biological experiments and a public human HSPCs dataset.
- SCALeBa improves the accuracy of differentiation trajectories in monocle2‐based pseudo‐time analysis.

## Graphical abstract

- Single‐cell transcriptome analysis with lentiviral barcoding (SCALeBa) to investigate the molecular characteristics of human HSPCs with different cell fates in vivo.
- Using SCALeBa, human HSPCs are divided into different subsets with signature genes identified.
- The legitimacy of identified genes with SCALeBa was validated using biological experiments and a public dataset.
- SCALeBa improves the accuracy of differentiation trajectories in monocle2‐based pseudo‐time analysis.

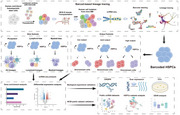

## 1 ARTICLE SUMMARY

Junnan Hua et al. developed a technology that combines single‐cell transcriptome Analysis with lentiviral barcoding (SCALeBa) to investigate the molecular characteristics of human hematopoietic stem and progenitor cells (HSPCs) with different cell fates in vivo. Using SCALeBa and in vivo transplantation, HSPCs are divided into several subsets according to their stemness and differentiation bias, with the molecular characteristics of these subsets and the gene‐level explanation for the heterogeneity of HSPCs being identified.

## 2 INTRODUCTION

Hematopoietic stem and progenitor cells (HSPCs) are the foundation of the adult hematopoietic system, playing a pivotal role in the long‐term maintenance and continuous production of all mature blood cell lineages throughout the lifespan of an organism.^1^ They serve as an exemplary model for studying stem cell biology. Concurrently, HSPCs‐based allogeneic stem transplantation has been extensively employed in clinical settings for the treatment of various haematological malignancies and genetic blood disorders, including transfusion‐dependent β thalassemia and congenital immunodeficiency.^2^ Therefore, understanding the molecular mechanisms underlying HPSC maintenance and differentiation is important for both fundamental scientific research and clinical applications.

Previous studies have demonstrated that HSPCs are functionally heterogeneous cells, including various cell subsets. Based on their self‐renewal capacity and differentiation potency, HSPCs can be categorized into long‐term hematopoietic stem cells (LT‐HSCs), short‐term hematopoietic stem cells (ST‐HSCs), multipotent progenitors (MPPs), common myeloid progenitors (CMPs), and common lymphoid progenitors (CLPs).^3^, ^4^ Recent advances in single‐cell RNA sequencing (scRNA‐seq) technology have facilitated the analysis of HSPCs at the single‐cell level, significantly enhancing our comprehension of HSPCs production, maintenance, and differentiation.^5^ To elucidate the molecular mechanisms governing the functional subsets within HSPCs, research scientists have utilized well‐characterized cell surface markers to separate HSPCs into distinct subsets with different potentials, and subsequently conducted scRNA‐seq to generate molecular signature maps of subsets of HSPCs at the single‐cell resolution.^5^, ^6^, ^7^ However, performing single‐cell sorting and scRNA‐seq based on a limited set of reported surface markers, may compromise the data accuracy and the comprehensiveness of analysis, as the subset cells with the same surface markers are still heterogenous which have been proved by scRNA‐seq. Another strategy is to conduct scRNA‐seq of HSPCs without cell sorting, and then define the HSPCs subsets based on the expression pattern at the single‐cell level.^8^ Unsupervised clustering is widely applied in single‐cell RNA‐sequencing (scRNA‐seq) to detect distinct cell clusters that can be annotated as known cell lineages or novel ones. However, it is always challenging to truly characterize the biological function of cell clusters identified by scRNA‐seq. This makes the reliability of such annotations questionable.^9^, ^10^

If the RNA expression profiles of HSPC subsets can be linked with their corresponding in vivo differentiation potential at the single‐cell level, it would greatly enhance our understanding of the molecular and regulatory mechanisms driving the diverse differentiation capacities of these subsets. These insights would provide a crucial theoretical foundation for utilizing and manipulating HSPCs in the treatment of various diseases.^11^ Inspired by T cells and TCRs if a random barcode sequence is added to the 3′UTR of an exogenous gene carried by a lentivirus, it is possible to identify and track the cellular identity in successfully transduced stem cells and their progeny at the single‐cell level while obtaining the single‐cell RNA expression profiles. We refer to this novel technique as the single‐cell transcriptome analysis with lentiviral barcoding (SCALeBa). By developing and optimizing SCALeBa and combining it with in vivo experiments in mice, we can establish the correspondence between single‐cell expression profiles and the in vivo differentiation potential of various cellular subsets within human HSPCs. This will elucidate the molecular mechanisms underlying the functional heterogeneity of human HSPCs. The established SCALeBa technology will also serve as an important tool to advance stem cell research across various fields.

## 3 RESULTS

### 3.1 Characterization of human hematopoietic cells from NCG‐X mice 24 weeks after transplantation

**FIGURE 1:**
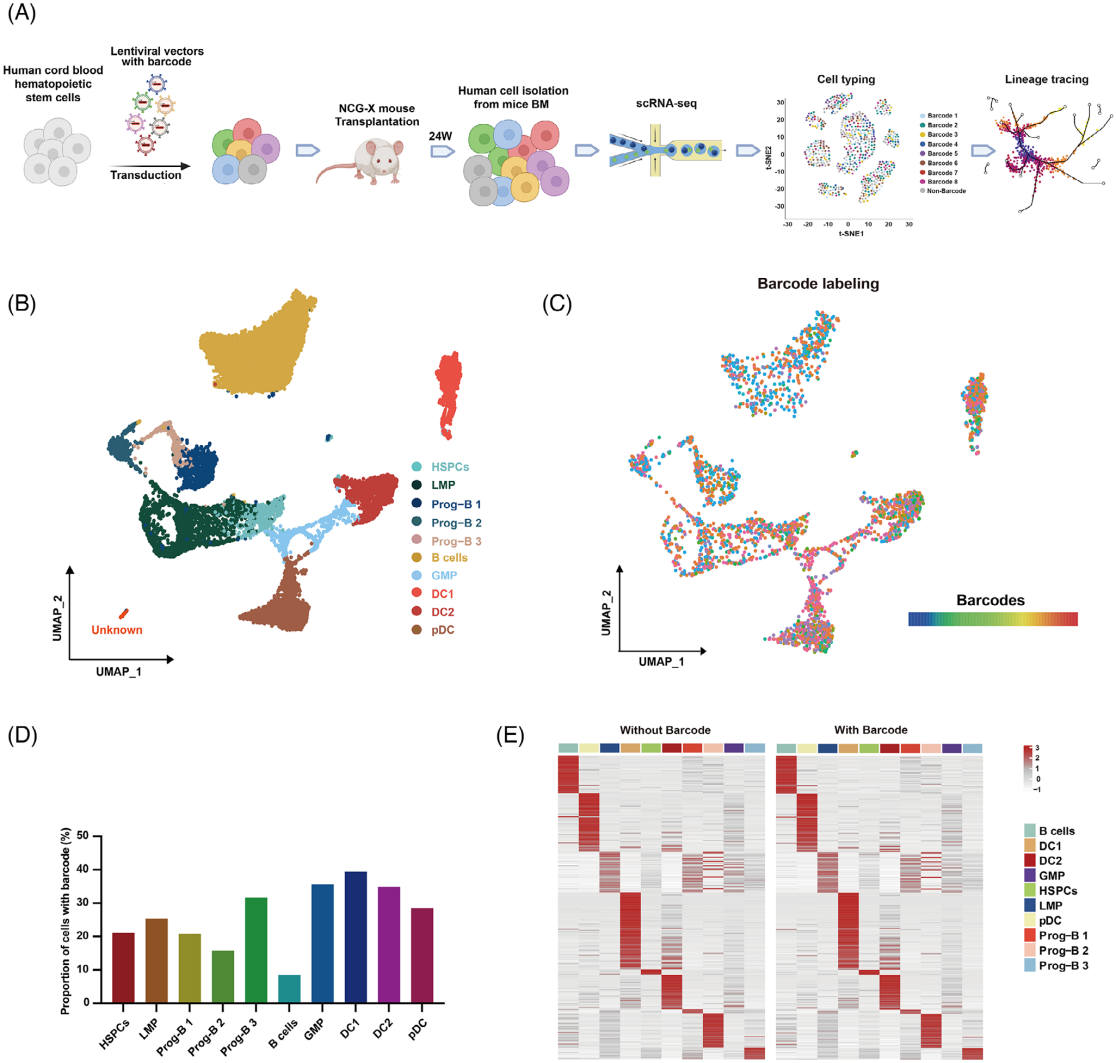
Characterization of human hematopoietic cells from NCG‐X mice 24 weeks after transplantation. (A) The diagram of using SCALeBa to characterize human HSPCs in vivo. Human CD34^+^ HSPCs from umbilical cord blood were transduced with SCALeBa lentiviruses that carry different barcodes in vitro. Subsequently, the transduced cells were transplanted into immunodeficient NCG‐X mice. After 24 weeks, bone marrow (BM) from these mice were collected and CD45‐positive human cells were isolated with magnetic beads and underwent single‐cell sequencing. Subsequent analysis for cell identity and barcode‐positive cells was conducted. (B) The UMAP visualization of the quality control passed 15 543 cells and ten cell lineages were classified. (C) The UMAP visualization of 3183 cells carrying 554 unique barcodes. Each colour represents one kind of barcode. (D) The proportion of barcode‐positive cells in each cell population. (E) Heatmap showing the expression levels of all genes in cell lineages with and without barcodes.

The single‐cell transcriptome analysis with lentiviral barcoding (SCALeBa) technology first utilizes a lentiviral library to insert random 20 bp barcodes into the genome of recipient cells. After conducting single‐cell sequencing on the cells, barcode extraction and analysis are performed to characterize the cell identity and then track the cell lineages. By examining the composition and distribution of barcodes in the downstream cells, information about the heterogeneity and differentiation bias of the upstream cells with the same barcodes can be obtained (Figure 1A and S1A). To verify the feasibility of SCALeBa technology, we confirmed the abundance of the barcode library is about 2.5 × 10^6^. Then approximately 5 × 10^4^ HSPCs from mobilized peripheral blood, a quantity less than one‐tenth of the library abundance, were transduced to ensure that most of the cells could carry unique barcodes after transduction. On the 12th day, uniform manifold approximation and projection (UMAP) from single‐cell sequencing showed barcode‐positive cells distributed across almost all subsets (Figure S1B,C), indicating that there was no apparent transduction bias. Meanwhile, the transduction bias values in different cell subsets were persistent from Day 4 to 7 (Figure S1D). Based on single‐cell transcriptome data and barcode analysis, the average transduction rate was 76.24% on the fourth day and 75.71% on the seventh day with lentiviral empty loading rates of 1.56% and 1.41%, respectively (Figure S1E). These results confirm that most cells were transduced and carried unique barcodes. Thus, lentiviral vector transduction of human HSPCs with SCALeBa resulted in efficient and specific transduction without affecting differentiation bias. These findings suggest that this technology can be used to transduce human HSPCs for mice transplantation. Then, we utilized SCALeBa to track the cell fate of HSPCs and to understand the corresponding molecular characteristics in vivo. CD34 positive  .8 × 10^5^ HSPCs derived from human umbilical cord blood were labelled with SCALeBa lentiviral library and transplanted into NCG‐X immunodeficient mice. After 24 weeks, BM samples from the mice were collected for human CD45^+^ cell isolation and single‐cell sequencing, and bioinformatic analysis was performed by retrieving and using the barcodes to predict cell activities and differentiations (Figure 1A). We captured a total of 22 493 cells, of which 15 543 passed quality control, with an average of 2509 genes and 8175 unique molecular identifiers (UMIs) per cell (Table S1). The cells with barcode and passed quality control is 3181, with an average of 3014 genes. Then, based on the feature gene expression of the main cell subsets projected onto UMAP, we divided the cell subsets into 10 categories, including HSPCs and other progenitor cells (Figure 1B). The accuracy of clustering of lymphoid and myeloid subsets was further verified by pseudo‐time series analysis (Figure S2) and expression pattern of marker genes (Figure S3). Similarly, we also applied UMAP to the subset of barcoded cells (Figure 1C) and the proportion of cells with barcode in each cell lineage were about 10%−40% (Figure 1D). Meanwhile, we found the expression levels of all genes were almost the same between cells with barcode and without barcode, suggesting the viral transduction did not affect the gene expression (Figure 1E).

### 3.2 Identification and characterization of human HSPCs with different output capabilities using SCALeBa

**FIGURE 2:**
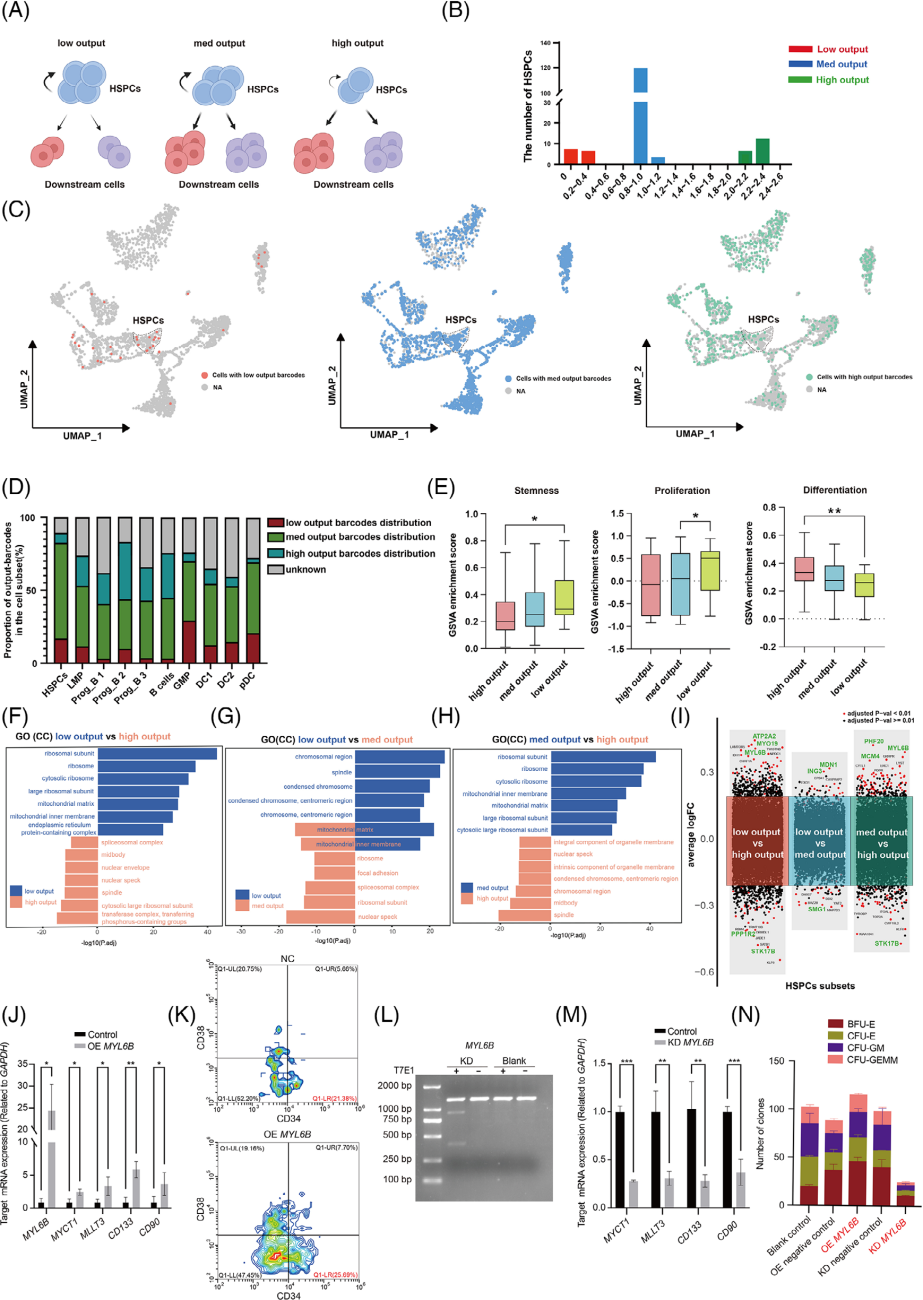
Identification and characterization of human HSPCs with different output capabilities using SCALeBa. (A) The schematic diagram shows the homeostasis and differentiation of HSPCs with low, med, and high outputs. The low‐output HSPCs exhibit more self‐renewal, the med‐output HSPCs maintain a balance between self‐renewal and downstream differentiation, and the high‐output HSPCs tend to downstream differentiation. (B) The output value of HSPCs is calculated by comparing the distribution ratio of cells with the same barcode between other cells and HSPCs. High outputs, value > 2.0, med outputs,  .8 < value < 1.2, and low outputs, value <.4. (C) The UMAP plot visually presents the distribution of HSPCs and their progeny cells with different output values. (D) The proportion of cells with different barcodes in barcode‐positive cells in each cell lineage. (E) The GSVA plot displays the scoring of the three output subsets in relation to the gene sets associated with stemness, proliferation (GO:0071425), and differentiation (GO:0060218). *p*‐value was calculated by *t*‐test, *p < .05; **p < .01. (F–H) The bidirectional gene enrichment plot for GO (Gene Ontology) CC (Cell Component) shows the enrichment of cellular components between the low, med, and high output HSPCs subsets. (I) The volcano plots showing the differential gene expression between low, med, and high output HSPCs subsets. The different subset‐related genes are shown in red and blue respectively in each panel. Key genes are highlighted in green. (J) The expression levels of *MYL6B* and other reported stemness genes were upregulated in HSPCs that were transduced with a lentiviral vector for the overexpression of *MYL6B*. The error bars are the SD. *p < .05; **p < .01. (K) The proportion of CD34^+^CD38^−^ cells was increased in HSPCs overexpressing *MYL6B*. (L) The *MYL6B* indels were induced by CRISPR/Cas9 editing, as determined by the T7 endonuclease assay. (M) The expression levels of reported stemness genes were decreased in HSPCs that were transduced with CRISPR/Cas targeting *MYL6B*. The error bars are the SD. *p < .05; **p < .01; ***p < .001. (N) CFU assay results of human HSPCs with overexpression or knockdown of *MYL6B*. *n* = 2, the error bars are the SEM.

To investigate the heterogeneity of HSPCs, we used barcoding to track the lineage of HSPCs and their downstream population. When a specific barcode is identified within a subset of HSPCs, we will count the fraction with the same barcode separately in the downstream and HSPCs subset and calculate the ratio of the two as the “output value”(Figure 2A). Through barcode tracing analysis, we found a total of 189 HSPCs have barcodes that also exist in the downstream population. Therefore, those HSPCs can be defined with output values and used for the following studies.

According to the distribution of all the output values, we found that those HSPCs were clearly divided into three subsets (Figure 2B). Therefore, we defined the HSPCs subset with output values of 0 to  .4 as the low‐output subset. It may indicate that HSPCs are more inclined towards self‐renewal. The output value between  .8 and 1.2 was defined as the med‐output subset. It may indicate that the downstream differentiation and self‐renewal ability of those HSPCs are roughly equal. The output value greater than 2.0 was defined as the high‐output subset. It indicates that the HSPCs represented by the barcode are more inclined to downstream differentiation. These three HSPCs subsets and their downstream cells with the corresponding barcodes were shown in the UMAP (Figure 2C), and the cells with median‐ and high‐output barcodes show a more widespread distribution when compared with cells with low‐output barcodes.

In HSPCs, the med and high output population account for more than 70% of the proportion, suggesting most HSPCs tend to differentiate. In agreement, in LMP and its downstream Prog B and B cells, as well as in GMP and downstream DC cells, there is a general trend that the proportion of cells with low, med output barcodes decreases along with differentiation (Figure 2D). The Gene Set Variation Analysis (GSVA) scores of the med and low output cell subsets are higher than those of the high output subset in gene sets related to stemness^12^ and proliferation,^13^, ^14^, ^15^, ^16^, ^17^, ^18^, ^19^, ^20^ while in differentiation‐related gene sets,^14^, ^21^, ^22^, ^23^, ^24^, ^25^, ^26^ the high output subset have higher scores (Figure 2E).

Gene enrichment analysis between the low‐ and high‐output populations revealed that genes highly expressed in the low‐output population were more enriched in ribosome‐related pathways, while genes highly expressed in the high‐output population were more enriched in nuclear and spindle‐related pathways. In Gene Set Enrichment Analysis (GSEA) differential analysis, the low‐output population was enriched DNA polymerase‐related signalling pathways, further indicating its stronger self‐replication and renewal ability (Figures 2F and S4A). When we compared the low‐output and med‐output populations, both of which have strong stemness, we found that they shared enrichment in mitochondrial matrix‐related pathways. It is well known that mitochondrial activity is one of the indicators of HSPCs stemness, and our results provide additional evidence for this viewpoint. GSEA results demonstrated that, compared with the med‐output population, the low‐output population enriched in pathways related to mitosis and spindle, indicating its slightly stronger self‐renewal ability than the med‐output population (Figures 2G and S4B). The gene enrichment analysis between the med‐output and high‐output populations showed that genes highly expressed in the med‐output population were enriched in ribosome‐related pathways, and GSEA results demonstrated that med‐output enriched in DNA replication‐related pathways. Those results suggest that the med‐output population, similar to low‐output populations, has higher stemness when compared with the high‐output population (Figures 2H and S4C).

Subsequently, we analyzed the differentially expressed genes between the three subsets to find the key factors that might contribute to the output differences of HSPCs. In the comparison between low output and high output, we found that genes such as *ATP2A2*, *MYL6B*, and *MYO19* were highly expressed in the low output subset, while genes like *PPP1R2* and *STK17B* were highly expressed in the high output subset (Figure 2I). The *ATP2A2* gene mainly functions in macroautophagy (Figure S4D). Comparing med output and high output revealed that genes such as *PHF20*, *MYL6B*, and *MCM4* were highly expressed in the med output subset, while genes like *STK17B* and *PPP1R2* were highly expressed in the high output subset (Figure 2I). The *PHF20* gene functions in histone modification and the *MCM4* gene functions in DNA replication and double‐strand break repair (Figure S4). Lastly, in comparing the low output subset with the med output subset, genes like *MDN1* and *ING3* were highly expressed in the low output subset, while genes like *SMG1* were highly expressed in the med output subset (Figure 2I). The *MDN1* gene functions in ribosome biogenesis and nuclear transport‐related pathways, *ING3* plays a role in DNA damage repair, and the *SMG1* gene functions in nuclear transport‐related pathways (Figure S4F). In conclusion, the high output subset exhibited high expression of the *PPP1R2* and *STK17B* genes in comparison with both the low output and med output subsets, and these genes are referred to as LS (low‐stemness) genes. In pairwise comparisons, we identified genes such as *ATP2A2*, *MYL6B*, *MYO19*, *PHF20*, *MCM4*, *MDN1*, and *ING3* that may be associated with stronger stemness, and these genes are referred to as HS (high‐stemness) genes.

Moreover, we experimentally verified the role of the above representative genes on HSPCs stemness. We constructed lentiviral vectors to transduce human CD34^+^ HSPCs for overexpression and knockdown of the corresponding genes. We first focused on *MYL6B*, as it was both upregulated in low and med output HSPCs subsets. After being transduced with a lentiviral vector overexpressing *MYL6B*, the HSPCs showed a significant upregulation of the *MYL6B* gene, as well as several reported stemness‐related genes,^27^, ^28^, ^29^ including *MYCT1*, *MLLT3*, *CD133*, and *CD90* (Figure 2J). The flow cytometry results showed that HSPCs overexpressing *MYL6B* had an increased CD34^+^CD38^−^ ratio (Figure 2K). Meanwhile, when *MYL6B* was knockdown with CRISPR/Cas (Figure 2L), the stemness‐related genes were significantly downregulated in transduced HSPCs (Figure 2M). The colony‐forming unit (CFU) assay showed that the total number of four clones (CFU‐E, BFU‐E, CFU‐GEMM, and CFU‐GM), was increased after overexpression of *MYL6B*, but decreased after its knockdown (Figure 2N). Furthermore, we further confirmed that the knockdown of *ING3, MDN1, MYO19*, and *PHF20* in HSPCs cells, also led to the downregulation of stemness‐related genes and the decreased clone numbers in CFU assay (Figure S5).

### 3.3 Identification and characterization of human HPSCs with pluripotency using SCALeBa

**FIGURE 3:**
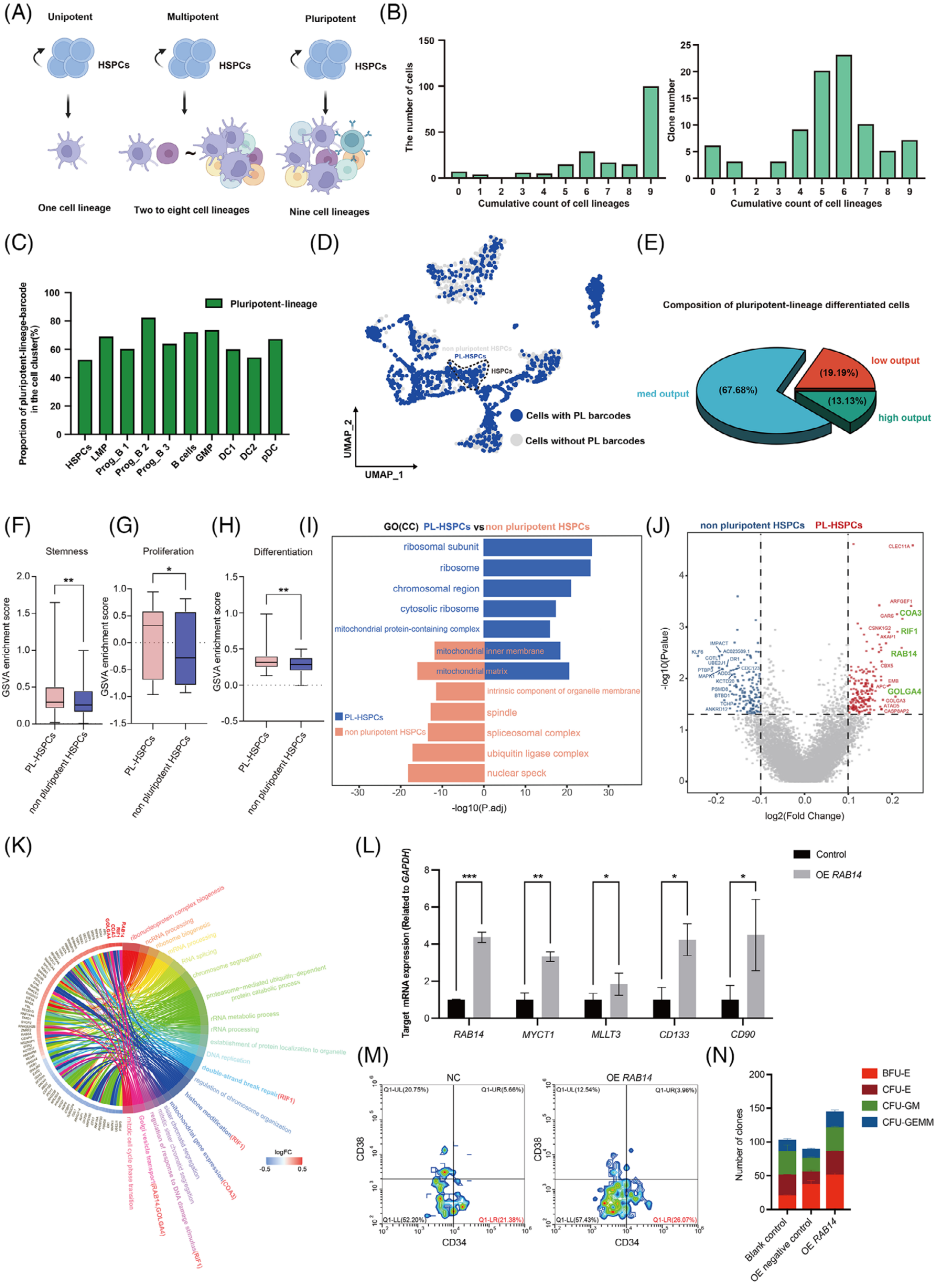
Identification of pluripotent HSPCs subset and its differential gene expression using SCALeBa. (A) The schematic diagram shows that the unipotent, multipotent, and pluripotent HPSCs subsets, which refer to the HSPCs that can differentiate into one cell lineage (*n* = 1), multiple cell lineages (*n* = 2∼8) and all cell lineages (*n* = 9), respectively. (B) The number of unique cell lineages for barcode distribution is denoted as the *M*‐value. An *M*‐value of 9 indicates the distribution of one barcode across all cell lineages. Additionally, the clone count corresponding to barcodes with different *M* values is also presented. (C) The bar chart displays the proportion of cells with pluripotent barcodes in barcode‐positive cells in each cell lineage. (D) The UMAP plot visually presents the pluripotent HSPCs subset and their progenies. (E) The Venn diagram shows the composition of pluripotent‐lineage HSPCs in terms of output values. (F–H) The GSVA plot displays the scoring of the PL‐HSPCs (pluripotent‐lineage) to non‐pluripotent HSPCs in relation to the gene sets associated with stemness, proliferation, and differentiation. *p*‐value was calculated by *t*‐test, *p < .05; **p < .01. (I) The GO(CC) bidirectional gene enrichment plot illustrates the enrichment of cellular component terms between the TL HSPCs and non‐pluripotent HSPCs. (J) The volcano plot illustrates the differential gene expression between the PL‐HSPCs and non‐pluripotent HSPCs. PL‐HSPCs and non‐pluripotent HSPCs‐related genes are shown in red and blue, respectively. Key genes are highlighted in green. (K) Circle plot illustrates the signalling pathways enriched for differentially expressed genes between the PL‐HSPCs and non‐pluripotent HSPCs subsets. (L) The expression levels of *RAB14* and other reported stemness genes were upregulated in HSPCs that were transduced with a lentiviral vector for the overexpression of *RAB14*. The error bars are the SD. *p < .05; **p < .01; ***p < .001. (M) The proportion of CD34^+^CD38^−^ cells was increased in HSPCs overexpressing *RAB14*. (N) CFU assay results of human HSPCs with overexpression of *RAB14*. *n* = 2. The error bars are the SEM.

Pluripotent differentiation ability is an important indicator of the stemness of HSPCs. Then we evaluated the pluripotent differentiation ability of the HSPCs using SCALeBa as a barcode‐based lineage tracing approach. The “M‐value” was calculated by counting the number of lineage types in which each HSPC barcode was distributed, ranging from 0 to 9. An M‐value of 9 indicates that the barcode carried by the HSPCs is distributed across all lineages, indicating that the HSPCs are capable of all lineage differentiation and pluripotent. In contrast, if the barcode is found in several but not all cell lineages or only in one certain cell lineage, it suggests the HSPCs carrying this barcode may be multipotent or unipotent with differentiation bias, and its *M*‐value should be less than 9 (Figure 3A). We found that a large proportion of barcoded HSPCs were pluripotent (Figure 3B) and thus we first focused on this subset. The relative distribution of barcodes corresponding to pluripotent HSPCs is approximately similar across all cell lineages (Figure 3C). In the UMAP plot, all cells with barcodes related to pluripotency are labelled, demonstrating that cells derived from the defined pluripotent HSPCs are distributed across all lineages (Figure 3D). Further analysis revealed that the majority of pluripotent HSPCs belong to the low‐med output subset, with only a small proportion belonging to the high‐output subset (Figure 3E).

To characterize the HSPCs with stronger self‐renewal capability, we selected pluripotent HSPCs that also belong to low and medium output subsets and defined these cells as PL‐HSPCs subsets. Then, we performed the gene expression analysis of this subset. Consistent with our speculation, the GSVA scores for stemness, proliferation, and differentiation‐related gene sets are higher in these PL‐HSPCs when compared with cells of non‐pluripotent HSPCs subsets (Figure 3F–H). Gene enrichment analysis suggested the PL‐HSPCs subset was enriched in the ribosome‐related pathway (Figure 3I), which is similar to those of the low output subset. The differential gene analysis showed that *COTL1, PTBP3*, and *KLF6* genes were downregulated, and *COA3*, *OLGA4*, *RIF1, and RAB14* were upregulated in the PL‐HSPCs subset, and these genes are referred to as pluripotency genes (Figure 3J). Interestingly, *KLF6* is also downregulated in low output HSPCs subset, suggesting its critical roles in both stemness and pluripotency. Further signalling pathway enrichment analysis of the upregulated genes in PL‐HSPCs revealed that the *RIF1* gene is enriched in DNA damage repair‐related pathways (Figure 3K), and DNA damage repair is crucial for maintaining the stemness and longer lifespan of hematopoietic stem cells.^30^

Moreover, we experimentally verified the effect of the *RAB14* gene on stemness. We constructed lentiviral vectors to transduce human CD34^+^ HSPCs for overexpression of the *RAB14* gene. After being transduced with a lentiviral vector overexpressing *RAB14*, the HSPCs showed a significant upregulation of the *RAB14* gene, as well as several reported stemness‐related genes,^27^, ^28^, ^29^ including *MYCT1*, *MLLT3*, *CD133*, and *CD90* (Figure 3L). The flow cytometry results showed that HSPCs overexpressing *RAB14* had an increased CD34^+^CD38^−^ ratio (Figure 3M). The CFU assay showed that four clones were increased after overexpression of *RAB14* (Figure 3N).

### 3.4 Identification and characterization of human HSPCs with biased differentiation using SCALeBa

**FIGURE 4:**
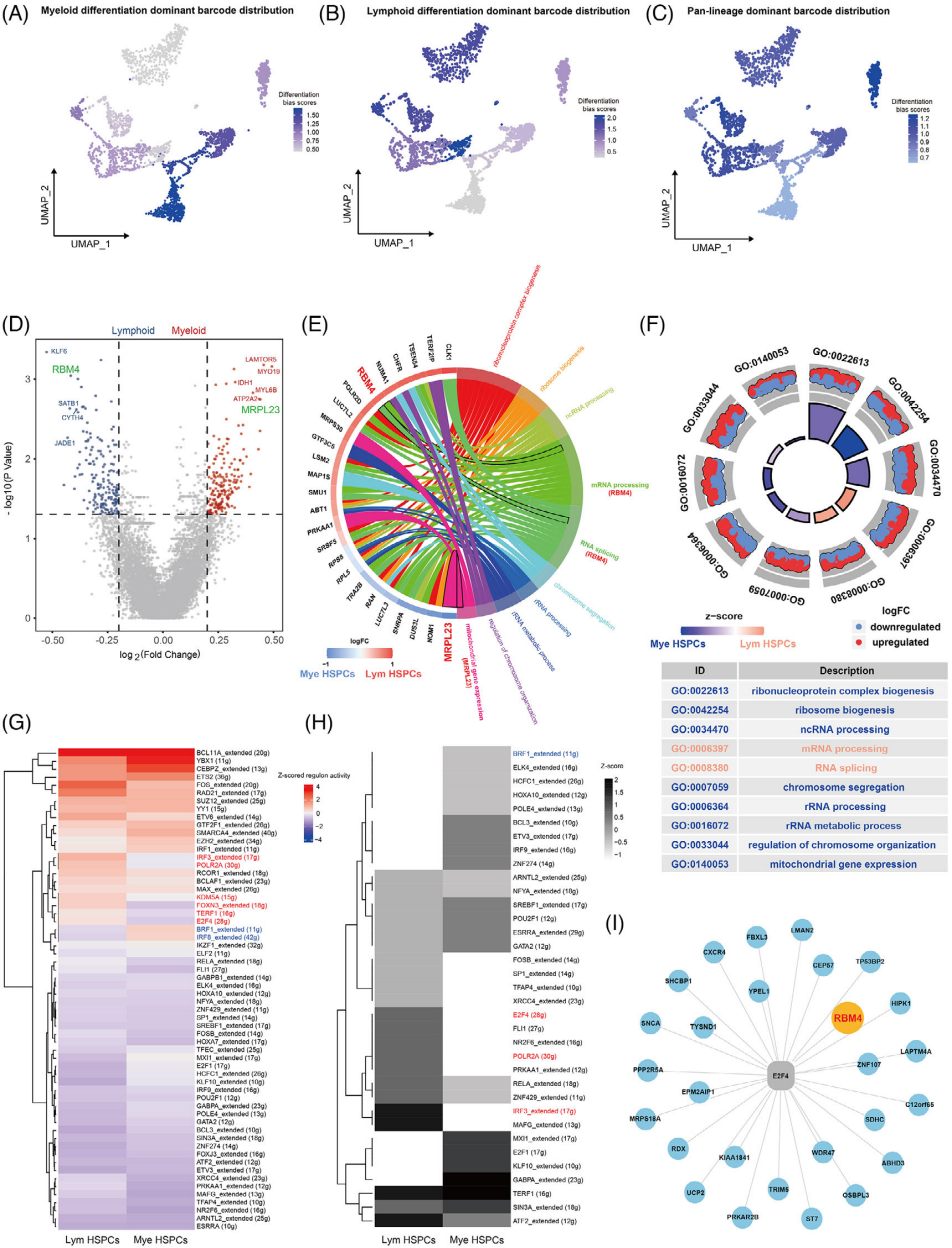
Identification and characterization of human HSPCs with biased differentiation using SCALeBa. (A–C) The UMAP plot visually presents the barcoded subsets with differentiation bias and pluripotency. (D) The volcano plot illustrates the differential gene expression between Mye and Lym HSPCs subsets. Mye HSPCs and Lym HSPC‐related genes are shown in red and blue, respectively. Key genes are highlighted in green. (E) Circle plot illustrates the signalling pathways enriched for differentially expressed genes between the Lym HSPCs and Mye HSPCs subsets. (F) GO enrichment circle diagram shows the signalling pathways enriched by the differential genes of Lym HSPCs and Mye HSPCs, and the GO term ID and description are shown in the table. (G) The AUC values of all cells in Lym HSPCs and Mye HSPCs were normalized for presentation. The colour keys from blue to red indicate AUC values from low to high. The names of regulons specifically upregulated in Lym HSPCs are shown in red, and the names of regulons upregulated in Mye HSPCs are shown in blue. (H) The binary regulon activity matrix was distributed and plotted as a heat map from the AUC of SCENIC, and the values of regulons in Lym HSPCs and Mye HSPCs were normalized. The dark colours in the diagram indicate the ON status of corresponding regulons. (I) The network diagram shows the genes regulated by the E2F4.

After characterizing the HSPCs subset with pluripotency and self‐renewal capabilities, we next focus on HSPCs subsets with a differentiation bias. Through barcode lineage analysis, we selected the representative barcodes that label HSPCs differentiating into lymphoid and myeloid lineages respectively. The cells with those barcodes were displayed on UMAP, showing clear distinct distribution patterns (Figure 4A,B). Additionally, we chose a clone with pan‐lineage differentiation as a control. Compared with the distribution of barcodes with differentiation bias, cells marked with pan‐lineage barcodes exhibited a more extensive distribution on the UMAP (Figure 4C). In consistency, these barcodes also show distinct enrichment patterns in corresponding progenies (Figure S6A).

To further understand the molecular mechanisms leading to differentiation bias, we analyzed the differentially expressed genes in HSPCs with differentiation bias. In the myeloid bias HSPCs subset (Mye HSPCs), genes such as *LAMTOR5*, *MYO19*, *MYL6B*, *IDH1*, *ATP2A2*, and *MRPL23* are highly expressed, while in the lymphoid differentiation bias HSPCs subset (Lym HSPCs), genes such as *KLF6*, *RBM4*, *SATB1*, *CYTH4*, and *JADE1* are highly expressed (Figure 4D). Further gene enrichment analysis revealed that the highly expressed *MRPL23* gene in Mye HSPCs is mainly enriched in mitochondrial gene expression‐related pathways, while the highly expressed *RBM4* gene in the Lym HSPCs is mainly enriched in mRNA processing and RNA splicing‐related signalling pathways (Figure 4E). It is worth mentioning that GSEA analysis confirmed that the Lym HSPCs were more enriched in immune response and activity‐related gene sets compared with the pluripotent HSPCs subset (Figure S6B). This further validates the differentiation heterogeneity within HSPCs and the practicality and accuracy of lineage tracing based on barcodes. Overall, enrichment analysis between these two subsets identified 10 significantly enriched signalling pathways, including the three signalling pathways involving the *MRPL23* and *RBM4* genes (Figure 4E,F). This confirms, to some extent, the role played by the *MRPL23* and *RBM4* genes and the signalling pathways they regulate in the differentiation bias of HSPCs. *MRPL23* and *RBM4* are referred to as myeloid differentiation bias (MDB) genes and lymphoid differentiation bias (LDB) genes, respectively.

To explore the differences in regulatory mechanisms between different differentiation bias subsets, we conducted SCENIC TF analysis on Mye and Lym HSPCs. In the Mye HSPCs subset, *BRF1* and *IRF8* regulons showed relatively high AUC values, while in the Lym HSPCs subset, regulons such as *E2F4*, *POLR2A*, and *IRF3* exhibited relatively high AUC values (Figure 4G). From the perspective of TF binary regulon activity, among the TFs with high AUC values mentioned above, the *BRF1* TF in the Mye HSPCs subset was “on”, while the *E2F4*, *POLR2A*, and *IRF3* TFs in the Lym HSPCs subset were “on” (Figure 4H). Upon further investigation of the genes regulated by the TFs mentioned above, we found that the genes regulated by the *E2F4* TF specifically include the *RBM4* gene (Figure 4I), suggesting *E2F4‐RBM4* may play an important role in lymphoid differentiation.

### 3.5 The stemness, pluripotency, and differentiation‐bias genes identified with SCALeBa can be verified in another independent dataset

**FIGURE 5:**
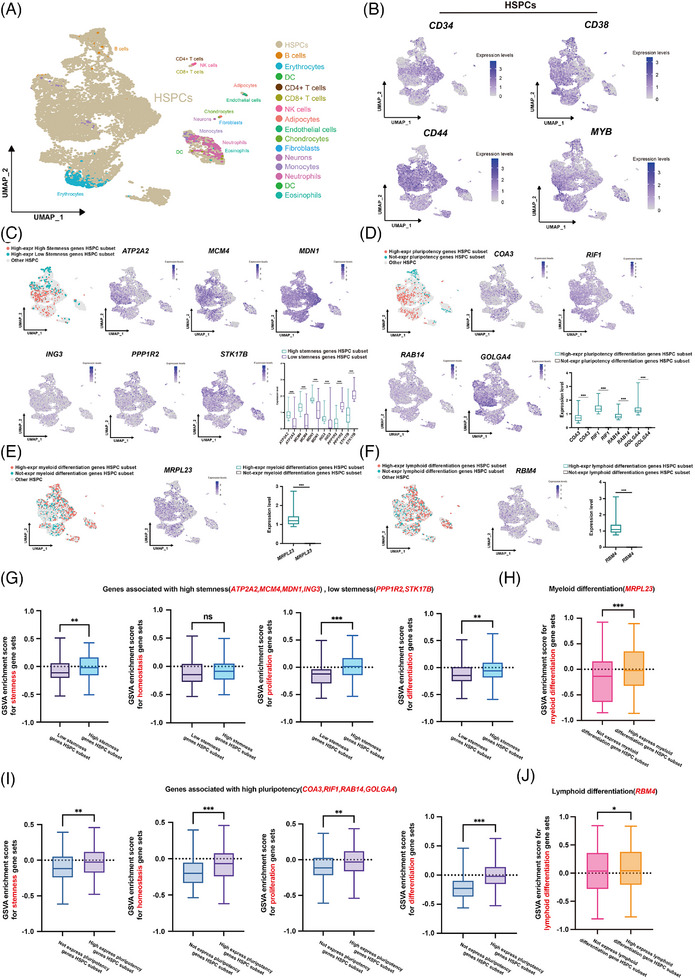
The stemness, pluripotency, and differentiation‐bias genes identified with SCALeBa can be verified in another independent dataset. (A) The UMAP visualization of 10776 Lin^−^CD34^+^cells passed the quality control, and 15 cell lineages were classified. Colour intensity indicates expression levels. (B) Expression of HSPCs signature genes were projected onto UMAP. Colour intensity indicates expression levels. (C) Subsets of HSPCs that exhibit high expression levels of high stemness genes (HS) or low stemness genes (LS) were labelled with different colours on the UMAP. The expression pattern of the signature genes of the two subsets was projected onto UMAP. Colour intensity indicates expression levels. *p*‐value was calculated by *t*‐test, **p < .01; ***p < .001. (D) Subsets of HSPCs that also highly/do not express pluripotency genes projected onto UMAP. Colour intensity indicates expression levels. The expression levels of the target genes of the two subsets are shown. *p*‐value was calculated by *t*‐test, ***p < .001. (E) Subsets of HSPCs that also highly and do not express MDB genes projected onto UMAP. Colour intensity indicates expression levels. On the right, the expression levels of the target genes of the two subsets are shown. *p*‐value was calculated by *t*‐test, ***p < .001. (F) Subsets of HSPCs that also highly and do not express LDB genes projected onto UMAP. Colour intensity indicates expression levels. On the right, the expression levels of the target genes of the two subsets are shown. *p*‐value was calculated by *t*‐test, ***p < .001. (G) The GSVA plot displays the scoring of the highly expressed HS/LS genes HSPCs subsets in relation to the gene sets associated with stemness, homeostasis, proliferation, and differentiation. *p*‐value was calculated by *t*‐test, **p < .01; ***p < .001. (H) The GSVA plot displays the scoring of the highly or not expressed pluripotency genes HSPCs subsets in relation to the gene sets associated with stemness, homeostasis, proliferation, and differentiation. *p*‐value was calculated by *t*‐test, ***p < .001. (I) The GSVA plot displays the scoring of the highly or not expressed MDB genes HSPCs subsets in relation to the gene sets associated with myeloid differentiation. *p*‐value was calculated by *t*‐test, **p < .01; ***p < .001. (J) The GSVA plot displays the scoring of the highly or not expressed LDB genes HSPCs subsets in relation to the gene sets associated with lymphoid differentiation. *p*‐value was calculated by *t*‐test, *p < .05.

In order to validate the consistency of the stemness, pluripotency, and differentiation bias‐related genes identified by SCALeBa with findings from other single‐cell transcriptome datasets, we conducted an analysis on a dataset consisting of 10 776 Lin^−^CD34^+^ cells obtained from healthy controls, transplant patients, and their grafts.^31^ After clustering, annotation, and further analysis, we specifically focused on 9844 cells annotated as HSPCs (Figure 5A,B).

Initially, we divided the 9844 HSPCs into subsets based on stemness‐related genes that had been identified with SCALeBa in our study. We identified subsets that exhibited high expression of HS‐ and LS genes, respectively (Figure 5C). Similarly, based on the pluripotency‐related genes identified with SCALeBa, we distinguished subsets that showed high expression of these pluripotency genes from those that did not (Figure 5D). Using the previously identified myeloid and lymphoid differentiation bias genes, we categorized HSPCs subsets into those with high expression of MDB/LDB genes and those that did not (Figure 5E,F).

Subsequently, we used sets of stemness,^12^ homeostasis,^32^, ^33^ proliferation,^13^, ^14^, ^15^, ^16^, ^17^, ^18^, ^19^, ^20^ and differentiation genes^14^, ^21^, ^22^, ^23^, ^24^, ^25^, ^26^ to perform GSVA scoring on these HSPCs subsets categorized by the aforementioned gene sets. The results indicated that subsets with high expression of all HS genes exhibited higher scores for stemness, proliferation, and differentiation‐related genes, except for homeostasis, which aligned with our expectations (Figure 5G). Similarly, the subsets with high expression of all pluripotency genes scored higher in stemness, homeostasis, proliferation, and differentiation‐related gene sets, which was fully consistent with our expectations as well (Figure 5H). For the HSPCs subsets with high and non‐expression of MDB/LDB genes, we used authoritative myeloid/lymphoid differentiation gene sets (GO:0045639, GO:1905458) for scoring. The results were as expected: HSPCs subsets with expression of MDB showed higher score in myeloid differentiation gene sets (Figure 5I), and HSPCs subsets with expression of LDB showed higher score in lymphoid differentiation gene sets than those without expression (Figure 5J).

In summary, we conducted a digital validation study to assess the performance of stemness, pluripotency, and differentiation bias‐associated genes identified using SCALeBa. Using another independent single‐cell transcriptome dataset, we confirmed that the HSPCs subsets characterized with our specific gene sets display the corresponding molecular signatures, suggesting the legitimacy of genes identified with SCALeBa.

### 3.6 SCALeBa application in pseudo‐time analysis using monocle2

Conventional pseudo‐time algorithms, in the absence of time‐series data, infer cell developmental paths based on gene expression patterns. This is predicated on the assumption that cells exhibit continuous changes in gene expression throughout their development.^34^ Therefore, we hypothesized that more accurate cell differentiation trajectories could be delineated by combining barcode tracing technology.

**FIGURE 6:**
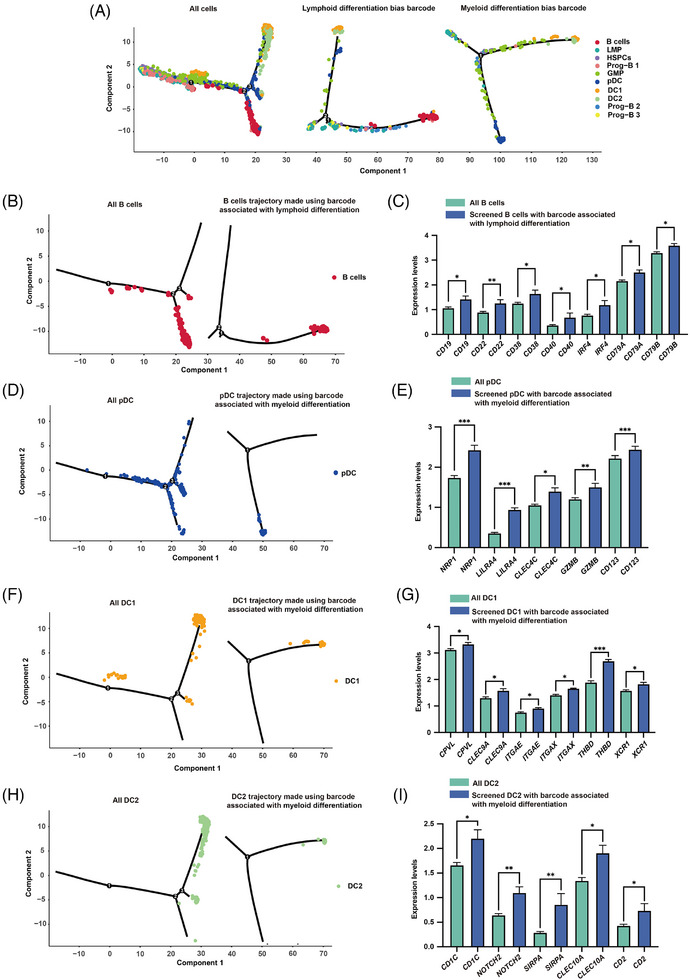
SCALeBa application in pseudo‐time analysis using monocle2. (A) Based on the pseudo‐time analysis plot using monocle2, it shows the differentiation trajectory using all cells, cells with lymphoid bias barcode and cells with myeloid bias barcode. The cell populations are labelled in different colours. (B) Using pseudo‐time analysis plots based on monocle2, the localization of B cells on trajectory was shown using all cell data, as well as cells with the lymphoid bias barcode. (C) Bar graph shows the difference in *CD19, CD22, CD38, CD40, IRF4, CD79A*, and *CD79B* genes expression between Lym bias B cells and all B cells (Lym bias barcoded B cell was eliminated). Unpaired *t*‐tests were used. n_Lym bias B _= 67, n_all B cell _= 5536, *p < .05; **p < .01. The error bars are the SEM. (D) Using pseudo‐time analysis plots based on monocle2, the localization of pDC cells on trajectory was shown using all cell data, as well as cells with the myeloid bias barcode. (E) Bar graph shows the difference in *NRP1, LILRA4, CLEC4C, GZMB* and *CD123* gene expression between Mye bias pDC and all pDC (Mye bias barcoded pDC was eliminated). Unpaired *t*‐tests were used. n_Mye bias pDC _= 70, n_all pDC _= 1950, *p < .05; **p < .01; ***p < .001. The error bars are the SEM. (F) Using pseudo‐time analysis plots based on monocle2, the localization of DC1 cells on trajectory was shown using all cell data, as well as cells with the myeloid bias barcode. (G) Bar graph shows the difference in *CPVL, CLEC9A, ITGAE, ITGAX, THBD*, and *XCR1* gene expression between Mye bias DC1 and all DC1 (Mye bias barcoded DC1 was eliminated). Unpaired *t*‐tests were used. n_Mye bias DC1 _= 47, n_all DC1 _= 890, *p < .05; ***p < .001. The error bars are the SEM. (H) Using pseudo‐time analysis plots based on monocle2, the localization of DC2 cells on trajectory was shown using all cell data, as well as cells with the myeloid bias barcode. (I) Bar graph shows the difference in *CD1C, NOTCH2, SIRPA, CLEC10A*, and *CD2* gene expression between Mye bias DC2 and all DC2 (Mye bias barcoded DC2 was eliminated). Unpaired *t*‐tests were used. n_Mye bias DC2 _= 25, n_all DC2 _= 701, *p < .05; **p < .01. The error bars are the SEM.

We utilized monocle2 to plot cell differentiation trajectories for all cells, as well as for cells with barcodes of lymphoid and myeloid biases, respectively (Figure 6A). Then, we separately compared the cell trajectories of several terminally differentiated lymphoid and myeloid cell lineages that are supposed to be at the end of the differentiation trajectory. B cell locations were more concentrated at terminal ends in the trace plotted using cells with the lymphoid bias barcode, as compared with the trace plotted using all cell data (Figure 6B). Further examination showed that the B cell marker genes such as *CD19, CD22, CD38, CD40, IRF4, CD79A*, and *CD79B* were expressed at a higher level in the B cells with lymphoid bias barcodes when compared with that of all other B cells (excluding barcoded B cells) (Figure 6C). Similarly, pDC locations were more concentrated at terminal ends in the trace plotted using cells with the myeloid bias barcode, as compared with the trace plotted using all cell data (Figure 6D). The marker genes such as *NRP1, LILRA4, CLEC4C, GZMB*, and *CD123* were expressed at a higher level in barcoded pDC when compared with all other pDC (Figure 6E). In the other two myeloid terminal differentiation subsets (DC1 and DC2), after redrawing with the cell myeloid bias barcodes, the DC differentiation trajectories were all at the end of the trajectories, and the DC marker genes of the two DC subsets, including *CPVL, CLEC9A, ITGAE, ITGAX, THBD, XCR1* and *CD1C, NOTCH2, SIRPA, CLEC10A, CD2* were expressed at a higher level in these barcoded DCs when compared with all other DCs (excluding DC with myeloid bias barcodes) (Figure 6F–I).

## 4 MATERIALS AND METHODS

### 4.1 Enrichment of CD34^+^ cells from human CB and mPB samples

Human CB and mPB samples were obtained with informed consent from a health donor (Shenzhen Children's Hospital). Mononuclear cells (MNC) were obtained by centrifugation on Lymphoprep medium, and MNC was enriched for CD34^+^ cell selection with the CD34 Microbead kit and LS column using MACS magnet technology (Miltenyi). The sorted CD34^+^ cells were subjected to downstream experiments.

### 4.2 Preparation of lentivirus with barcode

Based on the third‐generation lentiviral shuttle plasmid pCDH, the region between the long terminal repeat region (LTR) was modified, including replacing the promoter, adding GFP, and inserting the Nn Tag sequence at the 3 end of the gene and before the polyA signal. The Tag sequences were synthesized in vitro (*n* = 20), denatured, and annealed to form double strands, and then assembled with lentiviral shuttle plasmids through Gibson to obtain a lentiviral shuttle plasmid library carrying N20 Tag. The lentiviral shuttle plasmid library and helper plasmid were successfully constructed and co‐transfected into 293T packaging cells. The culture medium containing lentivirus particles was collected and concentrated.

### 4.3 Cell culture

For cell culture, CD34^+^ cells were resuspended in SCGM medium (Cellgenix) with the following recombinant hematopoietic cytokines: recombinant human stem cell factor (rhSCF) 100 ng/mL, recombinant human thrombopoietin (rhTPO) 100 ng/mL, recombinant human fms‐related tyrosine kinase‐3 ligand (rhFlt3‐L) 100 ng/mL. CD34^+^ cells were cultured in 24‐well tissue culture plates. The culture was maintained at 37°C in an atmosphere of 5% CO_2_ in an incubator (Thermo Fisher).

### 4.4 Lentiviral transduction

CD34^+^ cells were seeded into 24‐well plates at a density of 2−4 × 10^6^ cells/mL, with  5 mL of cell suspension per well. After a 24 h preactivation period, an equal volume of  .5 mL of transduction reagent was added to each well. The transduction reagent consisted of a viral solution mixed with a medium. Additionally, 100 ng/mL of poloxamer 407 and 100 ng/mL of dmPGE2 were incorporated into each mL of the transduction reagent. The culture medium was changed the day after transduction.

### 4.5 Animal guidelines

All animal procedures followed relevant guidelines and regulations. All protocols were approved and supervised by The Institutional Review Board of BGI.

### 4.6 Transplantation in mice

We used the immunodeficient mice model‐NCG‐X, with an age range of 4−6 weeks and female gender. A total of  .8 × 10^5^ cells were transplanted into the mice via tail vein injection. The mice were provided by GemPharmatech.

### 4.7 Bone marrow preparation and human CD45^+^ cell isolation

After euthanasia, bone marrow of NCG‐X mice was immediately isolated by flushing and crushing in 2% FBS‐PBS, and erythrocytes were removed with RBC lysis buffer. The mononuclear cells were then enriched for CD45^+^ cell selection using the CD45 Microbead kit and LS column using MACS magnet technology (Miltenyi).

### 4.8 scRNA‐seq

The DNBelab C Series High‐throughput Single‐Cell RNA Library Preparation Kit (MGI, #940‐000047‐00) was utilized to construct the sequencing libraries according to the manufacturer's protocol. In brief, single‐nucleus suspensions were used for droplet generation, emulsion breakage, beads collection, reverse transcription, second‐strand synthesis, cDNA amplification, and droplet index product amplification to generate barcoded libraries. The sequencing libraries were quantified by Qubit ssDNA Assay Kit (Thermo Fisher Scientific, #Q10212) and sequenced on the ultra‐high‐throughput DIPSEQ T1 or DIPSEQ T10 sequencers sequencer at the China National GeneBank.

### 4.9 Quality control of scRNA‐seq data

The DNBelab C Series HT scRNA analysis Software Suite (https://github.com/MGI‐tech‐bioinformatics/DNBelab_C_Series_HT_scRNA‐analysis‐software/tree/version1.0) was used for demultiplexing, barcode processing and single‐cell UMI counting. The software was applied with default parameters. The sequencing reads were paired‐end, with Read1 consisting of 30 bases. The first 20 bases of Read1 corresponded to cell barcodes, while the next 10 bases represented UMIs. Read2 contained 100‐bp cDNA sequences.

The processed reads were then aligned to the UCSC hg38 human genome using the STAR aligner with default settings.^35^ The resulting alignment files (SAM format) were converted to BAM format and annotated using a reference gene set with the help of PISA. UMIs within reads sharing the same cell barcode and gene annotation, and having a 1‐bp mismatch, were corrected to the most supported UMI. Gene‐cell metrics were generated to analyze valid cells, which were automatically identified based on the UMI number distribution for each cell. The Seurat R package (v.3.2.1)^36^ was employed for subsequent analysis. Quality control was performed using three indicators: the number of genes expressed per cell, the number of UMIs, and the proportion of mitochondrial RNA. Cells with abnormal gene expression (lower than Q1‐IQR or higher than Q3+IQR) were removed. Cells with a mitochondrial mRNA ratio greater than 10% were also excluded. Doublets were removed using the DoubletFinder R package (v.2.0.3).^37^

### 4.10 Dimensionality reduction and cell cluster

The Seurat (v.3.2.1) package was utilized to process the final cell‐gene matrix and create a Seurat object. This involved employing several functions in a sequential manner, namely “CreateSeuratObject”, “NormalizeData”, “FindVariableFeatures”, “ScaleData”, and “RunPCA”.^38^ The data were first normalized and scaled, followed by dimensionality reduction using principal component analysis (PCA). The top 40 significant principal components, which provide a compressed representation of the dataset, were identified using the “RunPCA” function. Subsequently, a graph‐based clustering method was employed to construct a shared nearest neighbour graph for the dataset. This was accomplished using the “FindNeighbors” function, which calculated the pairwise distances between cells. The modularity function was optimized to determine clusters, employing the “FindClusters” function with a resolution set to .6. Finally, the UMAP algorithm was utilized to learn the underlying manifold of the data and project the cells in a low‐dimensional space. This step aimed to group similar cells into clusters.

### 4.11 Cell type annotation

Cell type annotation was performed using marker genes obtained from the CellMarker 2.0 (http://bio‐bigdata.hrbmu.edu.cn/CellMarker/) and panglaoDB website (https://panglaodb.se/).

### 4.12 Identification and labelling of cells with barcodes

We developed a Perl program to capture barcodes from FASTQ files. It identifies barcodes through different patterns based on fixed upstream and downstream sequences.The program performs quality control on these barcodesand maps each one to a unique cellid. Finally, it annotates the barcode information into the Seurat object in subsequent analyses.

### 4.13 Output value analysis

We took each cell in the HSPCs as the research object, counted the number of cells with the same barcode in all non‐HSPCs cells, divided by the total number of cells in all non‐HSPCs cells, denoted as O1. Subsequently, we counted the number of cells with the barcode in the HSPCs, divided by the total number of cells in the HSPCs, denoted as O2. The ratio of O1 to O2 is referred to as the “output value”. Due to the accuracy of barcode tracing, we deleted HSPCs with more than three barcodes, and for HSPCs with two barcodes, we connected two barcodes into one barcode for tracing.

### 4.14 Pluripotency analysis

Taking each cell in the HSPCs as the research object, we counted the number of types of cells with the same barcodes in the downstream lineages as the M value. Since there are nine types of downstream cell lineages, the range of *M* value is 0−9. We define HSPCs with an M value of 1 as unipotent, those from 2 to 8 as multipotent, and 9 as pluripotent.

### 4.15 Lineage differentiation bias analysis

We took each cell in the HSPCs as the research object, counted the number of cells with a specific barcode in a downstream lineage and divided by the total number of cells in that lineage, denoted as D1. Next, we calculated the number of cells with the barcode in all lineages and divided by the total number of cells, denoted as D2. D1/D2 represents the differentiation bias score of the HSPCs subset with that specific barcode. After calculating the differentiation bias scores of all HSPCs, we identified HSPCs with lymphoid/myeloid lineage differentiation bias.

### 4.16 Differential gene expression analysis

We identified the DEGs differential gene expression by applying the “FindMarkers” function (Wilcoxon rank‐sum with *p*‐values for multiple testing with the Benjamini–Hochberg correction). The plot was generated using R software (v.4.2.2) package “ggpubr” (v0.4.0) and “ggplot2” (v3.4.2).

### 4.17 Gene enrichment analysis

Gene enrichment analysis was performed by using Gene Enrichment Analysis (GO database) tools in Hiplot Pro (https://hiplot.com.cn/), a comprehensive web service for biomedical data analysis and visualization. Terms with the *q* value < .05 were considered statistically significant.

### 4.18 GSEA analysis

GESA analysis was performed by using GSEA (GO database) tools in Hiplot Pro (https://hiplot.com.cn/), a comprehensive web service for biomedical data analysis and visualization.

### 4.19 GSVA Signature scoring

Assessing function among different cell subpopulations was conducted by scoring the genes related to stemness,^10^ homeostasis (GO:0061484), proliferation (GO:0071425), differentiation (GO:0060218) and myeloid/lymphoid differentiation gene sets (GO:0045639, GO:1905458) using GSVA. (https://www.bioconductor.org/packages/devel/bioc/vignettes/GSVA/inst/doc/GSVA.html). *p*‐value < .05 were considered statistically significant.

### 4.20 Pseudo‐time analysis

The single‐cell pseudotime trajectories were generated with the monocle2 package in R4.0.3.^39^ The newCellDataSet(), estimateSizeFactors(), and estimateDispersions() were used to perform these analyses.

### 4.21 Construction of knockdown and overexpression vectors

The CRISPR/Cas9 system was utilized for the construction of knockdown vectors. Guide RNAs (gRNAs) targeting the gene of interest were designed using online tools (https://chopchop.cbu.uib.no/). The gRNA sequences were cloned into a lentiCRISPRv2 vector, which co‐expresses Cas9 and the gRNA. The target sequences for gRNA were selected based on their efficiency and specificity scores. The knockdown vector included a puromycin resistance gene for the selection of stably transfected cells. For overexpression studies, the coding sequence (CDS) of the target genes was amplified and cloned into the lentiviral shuttle plasmid pCDH under the control of the EF1a promoter. The overexpression vector contains GFP, which can be used to identify stably transduced cells in flow cytometry. All the vectors were verified by Sanger sequencing to ensure correctly construction. The sequences of gRNA and primers are provided in Table S2.

### 4.22 Preparation of knockdown and overexpression lentiviral vectors

Lentiviral particles were produced by co‐transfecting the knockdown or overexpression vectors along with packaging plasmids (psPAX2 and pMD2.G) into 293T cells using jetOPTIMUS DNA transfection reagent. After 48 and 72 h, the supernatant containing lentivirus was collected, filtered through a  .45 µm filter membrane, concentrated, and stored. The titer of the virus was determined by transducing HEK293T cells at a gradient concentration.

### 4.23 Hematopoietic colony‐forming unit assay

For the CFU assay, 1000 HSCs transduced with lentivirus were plated in 35 mm Petri dishes using 1 mL of MethoCult H4435 medium. The dishes were incubated at 37°C in a humidified atmosphere with 5% CO_2_ for 14 days. After 14 days of incubation, colonies were scored using an inverted microscope. Colonies were classified as BFU‐E, CFU‐E, CFU‐GM, or CFU‐GEMM based on their morphology. The total number of colonies and the percentage of each colony type were calculated.

### 4.24 Flow cytometry analysis of HSPCs

HSCs transduced by lentivirus at day 10 were collected, washed in DPBS, and then incubated with fluorescence conjugated antibody CD34 (Biolegend) and CD38 (Biolegend) at 4°C for 30 min, washed and resuspended in DPBS for flow cytometry analysis. In the analysis, we selected cells expressing GFP, which were stably transduced by the lentiviral vector and overexpressed the gene of interest. The stemness of the cells was assessed by analyzing the proportion of CD34^+^CD38^−^ cells among GFP‐positive cells. Compensation was applied to correct for spectral overlap.

### 4.25 Puromycin selection of HSPCs transduced with knockdown lentivirus

The screening concentration of purinomycin was determined by pretest with gradient concentration. Fresh stem cell media were added to the cells containing puromycin at a final concentration of 2 µg/mL. The cells were then incubated at 37°C in a humidified atmosphere with 5% CO_2_. The media were changed every 2 days.

### 4.26 T7 Endonuclease I (T7E1) assay

After 48 h of puromycin selection, collect the cells and extract genomic DNA using Tiangen's reagent kit. Amplify the region of interest through PCR using PrimeStar GXL (Takara) and purify the PCR product using the NucleoSpin Gel and PCR clean‐up kit. 200 ng of the purified PCR product was digested with  .5 µL of T7E1 (New England Biolabs) at 37°C for 15 min. The sequences of PCR amplification primers are provided in Table S2.

### 4.27 Real‐time quantitative reverse transcription PCR for the detection of stemness genes

The total RNA was extracted by TRIzol (Invitrogen) following the manufacturer's protocol. We performed reverse transcription using the PrimeScript RT reagent Kit (Takara) according to the protocol. The PCR primer pairs’ sequences are provided in Table S2. The relative gene expression levels were calculated using the 2^−ΔΔCt^ method, normalizing to the housekeeping gene *GAPDH*. The efficiency of each primer pair was confirmed to be approximately equal to ensure accurate quantification. Statistical analysis was performed using Student's *t*‐test or one‐way ANOVA as appropriate. *p*‐value < .05 were accepted as statistically significant (*p‐value  < .05; **p‐value  < .01; ***p‐value  < .001).

## 5 DISCUSSION

SCALeBa presents a scRNA‐seq compatible lineage‐tracing methodology that diverges from traditional lineage‐tracing strategies,^40^ as it concurrently associates cell states with clonal fates from diverse initial conditions, eliminating the necessity to specifically target each progenitor state. It is an unbiased multilineage tracing technology. In our study, this technology enables the investigation of the molecular mechanisms underlying the heterogeneity of human HSPCs. Additionally, the SCALeBa lineage tracing technique proved to be a useful tool to correct pseudo‐time analysis results and address potential artefacts in the analysis process. In previous studies, researchers have developed a similar technology called LARRY to study the underlying mechanism of mice hematopoiesis and uncover *TCF5*’s critical role.^41^ Here, we independently developed SCALeBa with an abundance of lentiviral libraries reaching 2.5 × 10^6^, which is important for unique barcoding. Meanwhile, we applied this technology to the human HSPCs by overcoming the low transduction efficiency of human HSPCs.

By utilizing the SCALeBa technology, we gained valuable insights into the molecular mechanisms underlying the heterogeneity, stemness, and differentiation bias of human HSPCs. Our study confirms the previously discovered heterogeneity of HSPCs,^42^ which has significant implications for research related to hematopoietic stem cell diseases, clonal hematopoiesis, and ageing of HSPCs. Our analysis of low, med, and high output subsets showed that high expression of *MYL6B* genes may be related to high stemness, and their related mechanisms may be related to p53.^43^ The *MYL6B* gene has been reported to promote the development of HCC,^43^ and we further confirmed the role of *MYL6B* gene in the stemness of HSPCs by means of SCALeBa and in vitro experiments. In addition, *MYO19*, *ATP2A2*, *PHF20*, *MDN1*, *ING3*, and *MCM4*, which are highly expressed in stronger stemness subset, may also affect the stemness of HSPCs by acting on different pathways, such as ridging of the mitochondria cristae (*MYO19*), macroautophagy (*ATP2A2*), histone modification (*PHF20*, *ING3*), nuclear transport (*MDN1*), and DNA replication (*MCM4*). Previous studies have shown that *ING3* promotes prostate cancer growth by activating the androgen receptor,^44^ and *PHF20* promotes glioblastoma cell malignancies through a WISP1/BGN‐Dependent pathway.^45^ The mitochondria localized actin motor‐*MYO19* is critical for maintaining cristae structure, by associating with the SAM‐MICOS super complex.^46^ *MDN1* mutation is associated with a high tumour mutation burden and unfavourable prognosis in breast cancer.^47^ However, the effects of these aforementioned genes on the stemness of HSPCs are still unknown. We have confirmed the relationship between these genes and the stemness of HSPCs through SCALeBa technology and further validated their potential function in the maintenance of HSPCs stemness via cell culture experiments. At the same time, the high expression of *PPP1R2* and *STK17B* genes may be related to the low stemness of the high output subset. In the analysis of the pluripotent HSPCs subset, we found the high expression of previously reported stem cell growth factor *CLEC11A*.^48^ Meanwhile, two previously reported genes related to tumour invasion and migration, *RIF1* ^49^ and *RAB14*,^50^ are also highly expressed in PL‐HSPCs. Previous studies have shown that the *RAB14* gene can promote the development of bladder cancer and non‐small‐cell lung cancer.^50^, ^51^ Here we have shown the higher expression of *RAB14* in PL‐HSPCs by SCALeBa, and its overexpression results in increased CD34^+^CD38^−^ HPSCs ratio and colony number, suggesting its important roles in HSPCs. In addition, *COA3* and *GOLGA4*, which are highly expressed in the pluripotent‐lineage HSPCs subset, may also affect the pluripotent of HSPCs by acting on mitochondrial gene expression (*COA3*), Golgi vesicle transports (*GOLGA4*) pathways, respectively. Moreover, human HSPCs expressing *MRPL23* and *RBM4* genes demonstrate a tendency to differentiate into myeloid and lymphoid lineages respectively in vivo. Regulon analysis indicated that transcription factor E2F4, which was highly expressed in lym‐HSPCs, might regulate the downstream effector *RBM4*. This suggests that E2F4‐*RBM4* might play a critical role in lymphoid differentiation. Moreover, the legitimacy of the identified genes with SCALeBa was also validated using public human HSPCs dataset. In terms of methodology, our application of SCALeBa to pseudo‐time analysis using monocle2 has clarified the differentiation trajectories of several cell lines, particularly at the terminal end of differentiation. This advancement underscores the importance of considering potential artefacts in pseudo‐time analysis and the value of SCALeBa in refining such analytical approaches. The above findings may provide valuable insights into the broader principles of stem cell biology. We suggest that future studies should consider combining SCALeBa with other omics techniques to fully elucidate the complex regulatory networks in stem cells and their impact on disease and regeneration.

In the realm of HSPC research, the SCALeBa lineage‐tracing methodology stands as a beacon of promise, poised to transform our comprehension of the lineage commitment, heterogeneity, and differentiation processes that are pivotal in blood cell formation.^52^ This technology's capacity to refine and optimize its tracing capabilities will significantly enhance our ability to identify and monitor the developmental paths of these critical stem cells. The potential of SCALeBa is not limited to HSPCs; its application can be broadened to other stem cell fields, including those involved in organogenesis and induced pluripotent stem cells (iPSCs), where it can elucidate the complex processes of tissue regeneration and cellular reprogramming.^53^ Looking forward, the ongoing development of SCALeBa will likely integrate with other cutting‐edge single‐cell omics technologies, such as epigenomics, transcriptomics, proteomics, and metabolomics, to offer a multidimensional characterization of cells and further augment its analytical power and versatility.^40^, ^41^, ^53^, ^54^ This integration promises to reveal novel biomarkers and regulatory mechanisms, ultimately driving the advancement of innovative therapeutic strategies and reinforcing SCALeBa's role in the future of stem cell research and regenerative medicine.

## AUTHOR CONTRIBUTIONS

Chao Liu, Wenjie Ouyang, Sixi Liu, and Junnan Hua conceived and designed the study. Junnan Hua wrote the manuscript. Chao Liu, Ke Wang, Yecheng Xiong, Jiabin Ding, and Tingting Zhang contributed to the discussion and revision of the manuscript. Guoyi Dong, Yue Li, Sixi Liu, Xinru Zeng, and Wenjie Ouyang performed the experiments. Yuxi Li, Ying Gu, and Wenjie Ouyang participated in guiding and providing suggestions for the study. Junnan Hua, Haixi Sun, Xiaojing Xu, Yue Chen, and Rui Liu provided technical support and conducted data analysis. All authors read and approved the final manuscript.

## CONFLICT OF INTEREST STATEMENT

The authors declare no conflict of interest.

## ETHICS STATEMENT

This study was approved by the institutional review board of Shenzhen Children's Hospital and BGI. Written informed consent was obtained from all patients. All procedures were in accordance with the Declaration of Helsinki.

## Supporting information



Supporting Information

Supporting Information

## Data Availability

The scRNA‐seq data generated in this study have been deposited in the CNSA (https://db.cngb.org/cnsa/) of CNGBdb with accession code CNP0005534.
