# Supplementary material for: Molecular characterization of human HSPCs with different cell fates in vivo using single‐cell transcriptome analysis and lentiviral barcoding technology
Source: Clin Transl Med. 2024 Nov 13;14(11):e70085. doi: 10.1002/ctm2.70085 (PMC11560861; doi:10.1002/ctm2.70085)
Supplement: Supplementary file 1 — Supporting Information [file CTM2-14-e70085-s001.docx]

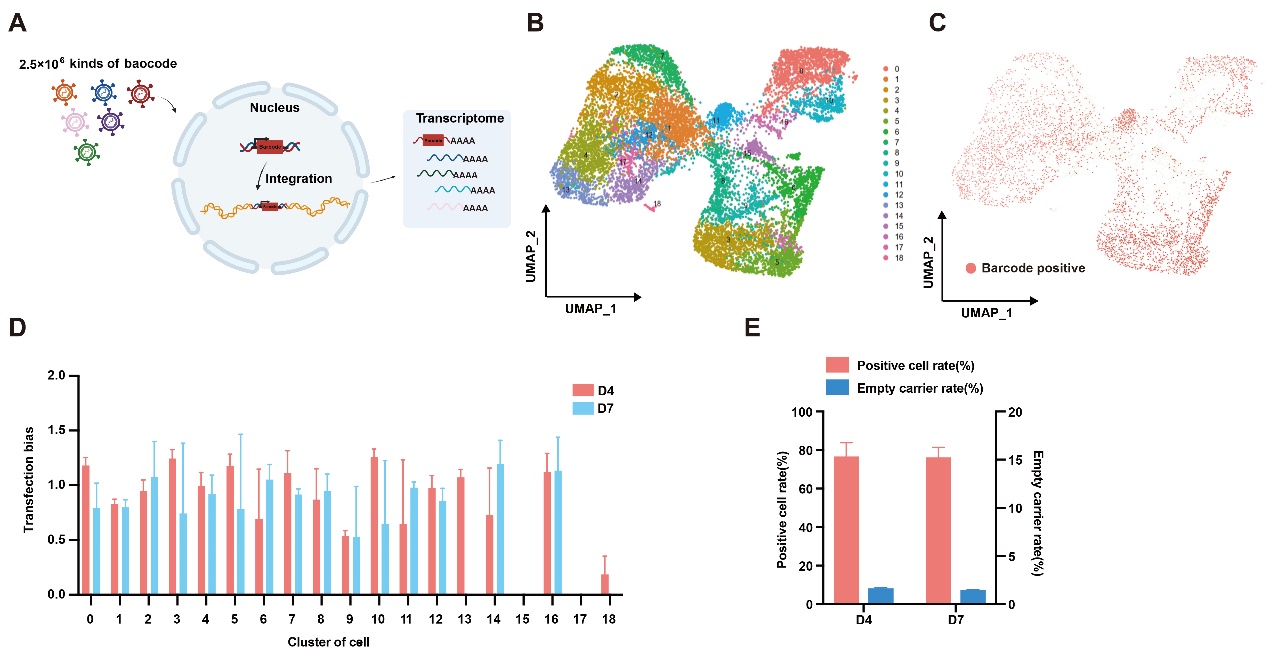


**Sup Fig1. Construction and quality validation of the lentiviral barcode library**

(A) Diagram illustrating the process of HSPCs transduction with barcode-carrying lentivirus.

(B, C) Representation of the distribution of barcode-positive cells in the UMAP plot on the twelfth day after in vitro transduction with lentivirus.

(D) Statistical analysis of the transfection bias of different subsets on the fourth and seventh days after *in vitro* lentivirus transduction (Transfection bias = Percentage of positive cells in a certain subset of cells in a certain sample (positivity rate of the subset) / Percentage of positive cells in a certain sample (positivity rate)).

(E) Statistical analysis of the transduction efficiency of lentivirus after *in vitro* transduction on the fourth and seventh days, as well as the rate of empty barcodes.


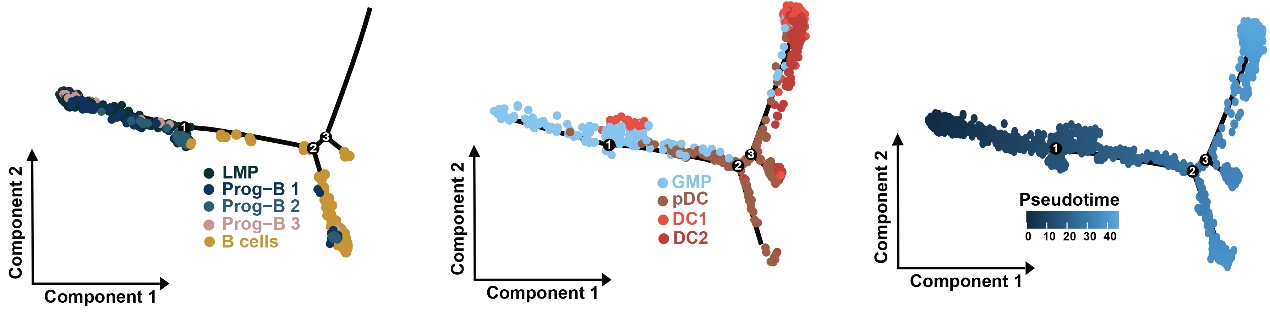


**Sup Fig2. Monocle2-based pseudo-time analysis**

Monocle2-based pseudo-time analysis plots showing LMP to B cells, GMP to pDC, DC differentiation trajectories, and pseudo-time results for all cells.


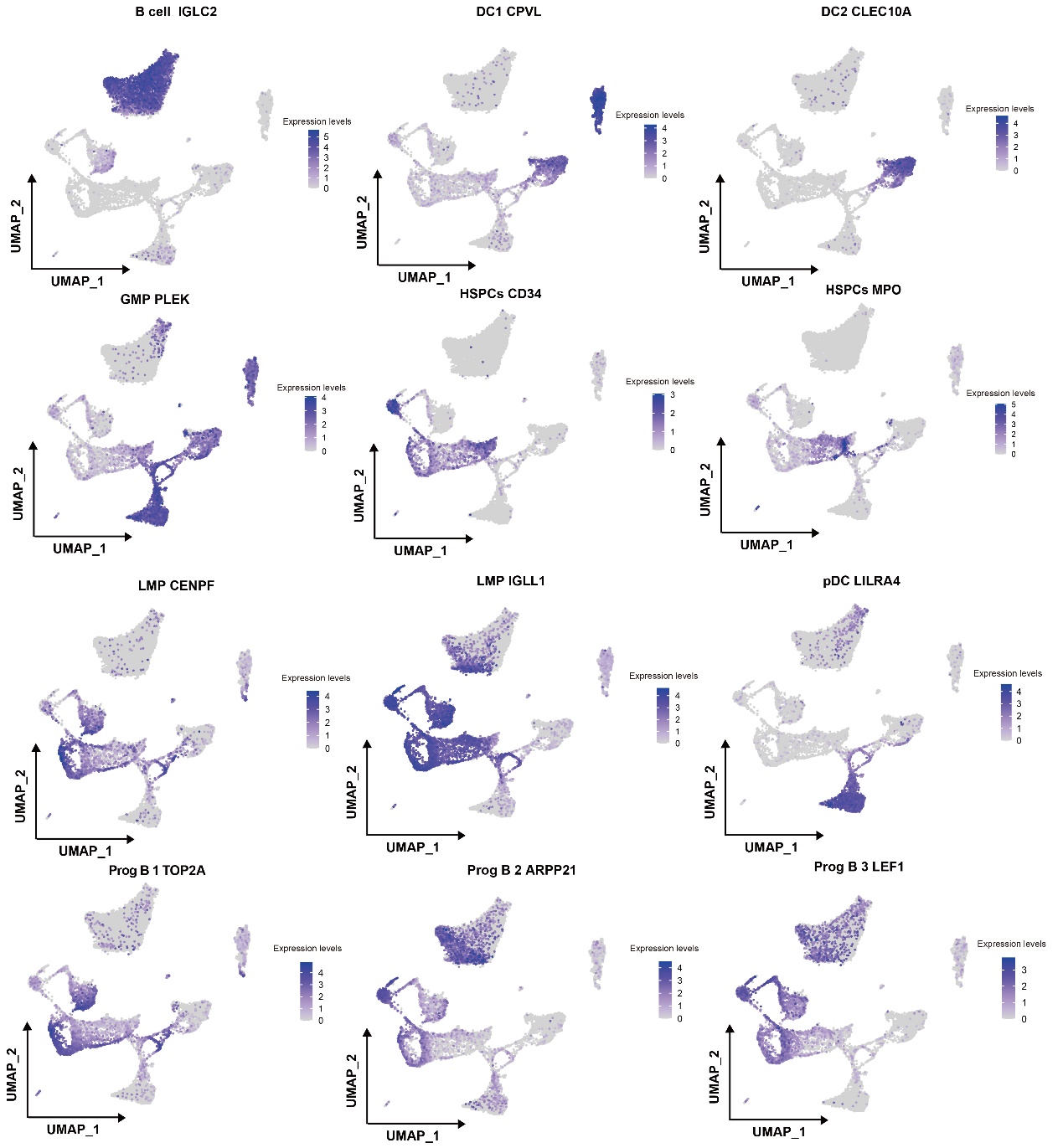


**Sup Fig3. Marker genes for cell lineages**

Expression of marker genes of the ten cell lineages projected onto UMAP. Color intensity indicates expression levels.


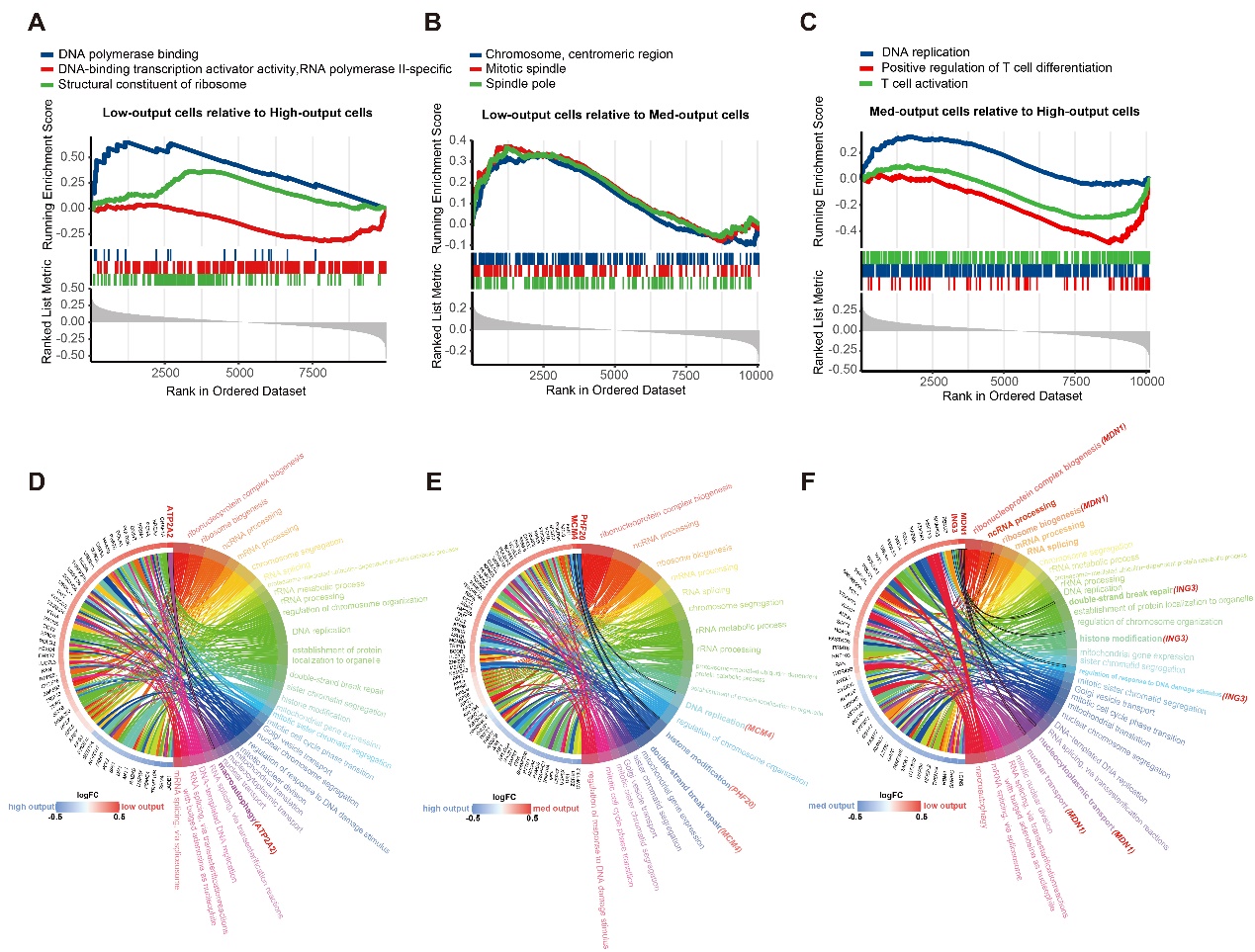


**Sup Fig 4. GSEA and enrichment analysis of high, med and low output subsets**

(A) GSEA analysis (GO) between low and high output subsets.

(B) GSEA analysis (GO) between low and med output subsets.

(C) GSEA analysis (GO) between med and high output subsets.

(D) Circle plot illustrates the signaling pathways enriched for differentially expressed genes between the low output and high output cells.

(E) Circle plot illustrates the signaling pathways enriched for differentially expressed genes between the med output and high output cells.

(F) Circle plot illustrates the signaling pathways enriched for differentially expressed genes between the low output and med output cells.


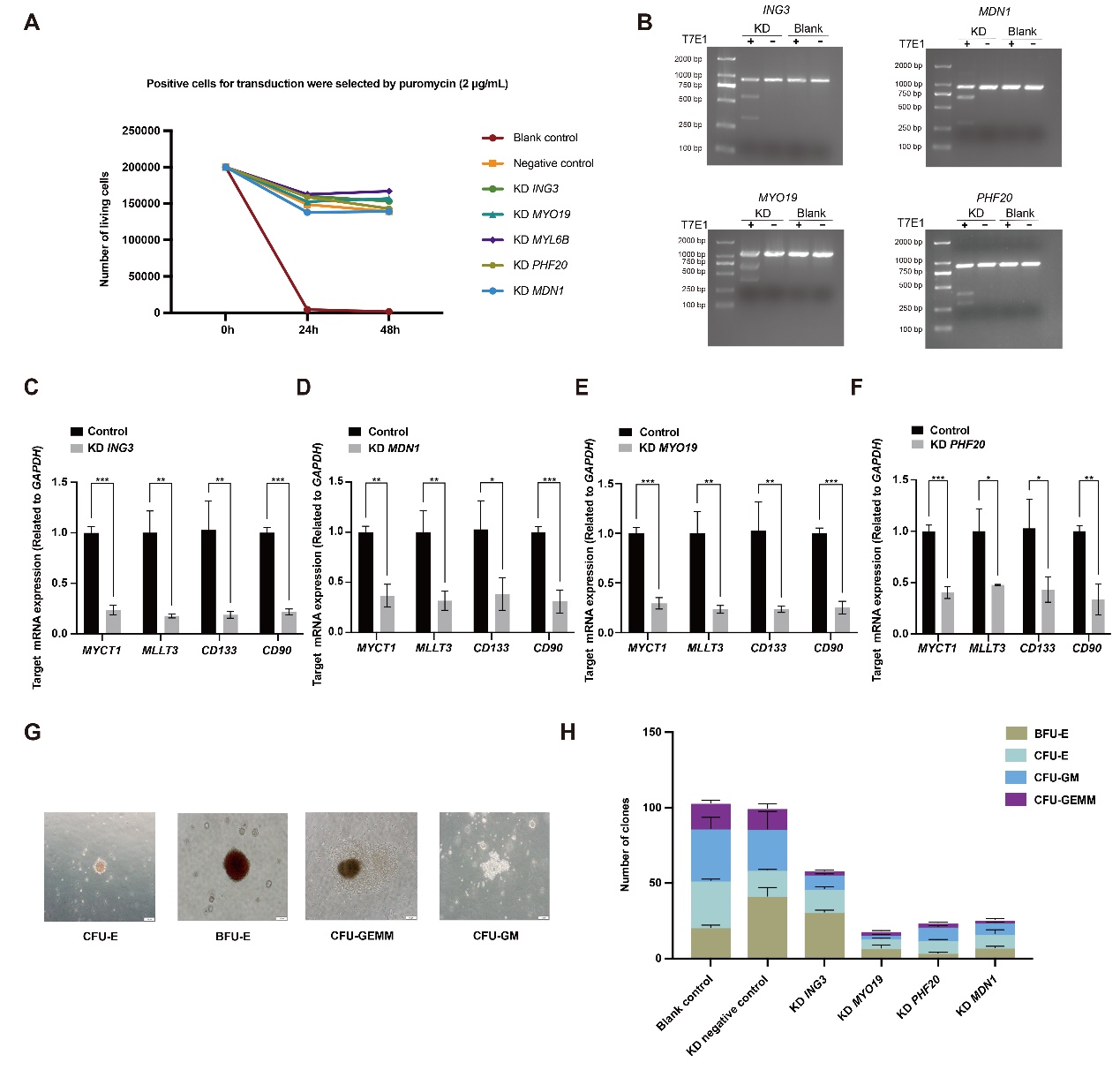


**Sup Fig 5. The roles of the genes identified using SCALeBa were validated using biological experiments**

1. The HSPCs transduced with lentivirus for gene knockdown were screened in the culture medium containing 2 μg/ml puromycin. The number of surviving cells at 24h and 48h in different groups was shown.

(B) The *ING3*, *MDN1*, *MYO19* and *PHF20* indels were induced by CRISPR/Cas9 editing, as determined by T7 endonuclease assay.

(C-F) The expression levels of reported stemness genes were decreased in HSPCs that were transduced with CRISPR/Cas targeting *ING3*, *MDN1*, *MYO19* and *PHF20*. The error bars are the SD. *P < 0.05; **P < 0.01; ***P < 0.001.

(G) Representative images of BFU-E, CFU-E, CFU-GM and CFU-GEMM colonies formed after 14 days of culture.

(H) CFU assay results of human HSPCs with knockdown of *ING3*, *MDN1*, *MYO19* and *PHF20*. n=2, the error bars are the SEM.


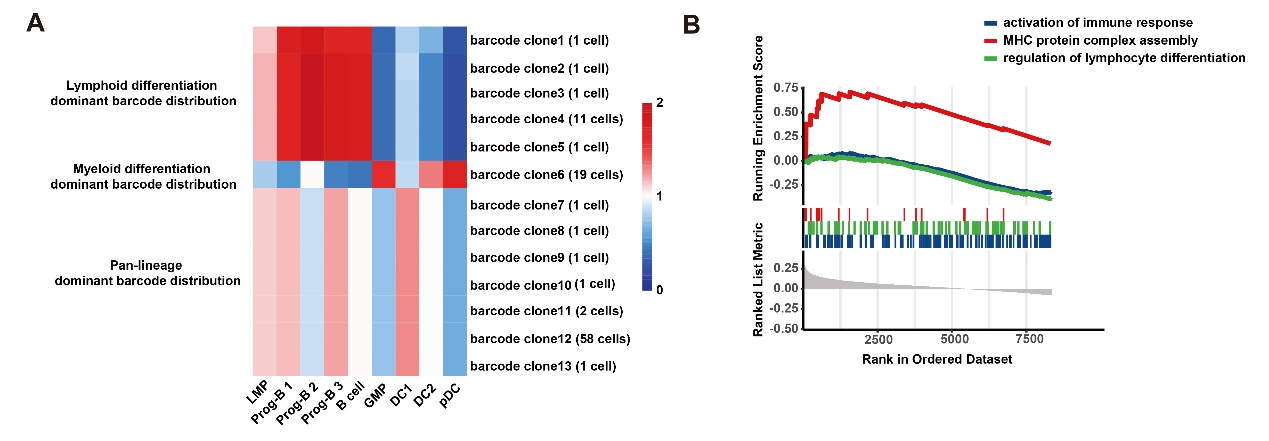


**Sup Fig 6. Selection and identification of lineage, myeloid, and pluripotent-lineage differentiation-biased subsets.**

1. The heatmap shows dominant barcode clones that are biased towards lymphoid, myeloid and pluripotent differentiation. The color intensity represents the proportion of barcode clones in the cell population, with darker colors indicating higher proportions in the subset.

(B) GSEA analysis (GO) between lymphoid and pluripotent-lineage bias subsets.
