# Supplementary material for: Molecular characterization of human HSPCs with different cell fates in vivo using single‐cell transcriptome analysis and lentiviral barcoding technology
Source: Clin Transl Med. 2024 Nov 13;14(11):e70085. doi: 10.1002/ctm2.70085 (PMC11560861; doi:10.1002/ctm2.70085)
Supplement: Supplementary file 2 — Supporting Information [file CTM2-14-e70085-s002.docx]

**Sup Table1. Quality of single cell sequencing data of NGG-X mouse bone marrow sample.**

|  | Estimated number of cell | Median UMI counts per cell | Median genes per cell | Mean reads per cell |
| --- | --- | --- | --- | --- |
| NCG-X-1^(BM)^ | 1060 | 11338 | 3108 | 195361 |
| NCG-X-2^(BM)^ | 753 | 13483 | 3584 | 248853 |
| NCG-X-3^(BM)^ | 1147 | 8122 | 2579 | 222493 |
| NCG-X-4^(BM)^ | 1331 | 10451 | 3059 | 221631 |
| NCG-X-5^(BM)^ | 1032 | 8744 | 2782 | 196333 |
| NCG-X-6^(BM)^ | 820 | 11471 | 3306 | 253275 |
| NCG-X-7^(BM)^ | 1041 | 11810 | 3365 | 230103 |
| NCG-X-8^(BM)^ | 1106 | 9204 | 2894 | 148575 |
| NCG-X-9^(BM)^ | 1130 | 10608 | 3092 | 209601 |
| NCG-X-10^(BM)^ | 3845 | 3121 | 1331 | 50699 |
| NCG-X-11^(BM)^ | 3349 | 2566 | 1154 | 45377 |
| NCG-X-12^(BM)^ | 2366 | 2948 | 1244 | 63013 |
| NCG-X-13^(BM)^ | 3513 | 2416 | 1119 | 37301 |

Note: NCG-X ^(BM)^ refer to NCG-X primary transplant mouse bone marrow sample.

**Sup Table2. The gRNA sequences and PCR amplification primer sequences used in this study.**

|  | sequence (5' To 3') |
| --- | --- |
| gRNA primer-*ING3*-F | CACCGTCTTCTAGGTACAACATCG |
| gRNA primer-*ING3*-R | AAACCGATGTTGTACCTAGAAGAC |
| gRNA primer-*MYO19*-F | CACCGACCGCGAAACCGACTGGAGC |
| gRNA primer-*MYO19*-R | AAACGCTCCAGTCGGTTTCGCGGTC |
| gRNA primer-*MYL6B*-F | CACCGCAACGGCTGCATCAACTACG |
| gRNA primer-*MYL6B*-R | AAACCGTAGTTGATGCAGCCGTTGC |
| gRNA primer-*PHF20*-F | AAACATTAAAACGTCCTCGGCTTGC |
| gRNA primer-*PHF20*-R | CACCGGGGGTTGACCGCATCGTCG |
| gRNA primer-*MDN1*-F | CACCGTGGGTCGAGTGCGGCTTCTA |
| gRNA primer-*MDN1*-R | AAACTAGAAGCCGCACTCGACCCAC |
| overexpression PCR primer-*MYL6B*-F | gagaatcccggccctATGCCTCCCAAGAAGGATGTTCC |
| overexpression PCR primer-*MYL6B*-R | CAGAGGTTGATTGTCGACTCAGACGCTTAGGATGTGTTTCAAGAAG |
| overexpression PCR primer-*RAB14*-F | gagaatcccggccctATGGCAACTGCACCATACAACTAC |
| overexpression PCR primer-*RAB14*-R | CAGAGGTTGATTGTCGACCTAGCAGCCACAGCCTTCTCT |
| T7E1 PCR primer-*ING3*-F | CGGGGCATGGAATCACATCA |
| T7E1 PCR primer-*ING3*-R | TATGCACTAGCGGAGTGAGC |
| T7E1 PCR primer-*MYO19*-F | GGAAGAAGTAGGCGTCTCCAC |
| T7E1 PCR primer-*MYO19*-R | GGACAATTGAGGCGGAAACAG |
| T7E1 PCR primer-*MYL6B*-F | GTTTAAAAAAAAGCAAAACAGCATGGGCTG |
| T7E1 PCR primer-*MYL6B*-R | CTTTATTTTGAAACAATAGGTGGCCTCCTG |
| T7E1 PCR primer-*PHF20*-F | GCGAGACTCCATCTCAAAAAAAAAAAATCCA |
| T7E1 PCR primer-*PHF20*-R | TTCTCAAACTCCTGACCTCAGATGATCC |
| T7E1 PCR primer-*MDN1*-F | CAGAGCAGTTTGCTTCTTTCACT |
| T7E1 PCR primer-*MDN1*-R | AGACATAAGCCTGACTTCCAACTG |
| qRT-PCR primer-*MYL6B*-F | ACCCCAAGAGTGATGAGCTG |
| qRT-PCR primer-*MYL6B*-R | CCTCATATGTGCCTTGGCCT |
| qRT-PCR primer-*RAB14*-F | TGCAGATTTGGGATACGGCA |
| qRT-PCR primer-*RAB14*-R | GCTCCGTGTAACAGCCCTAA |
| qRT-PCR primer-*GAPDH*-F | ACCCACTCCTCCACCTTTGA |
| qRT-PCR primer-*GAPDH*-R | CTGTTGCTGTAGCCAAATTCGT |
| qRT-PCR primer-*MLLT3*-F | AGCTTAAGTGATGGCAGCGA |
| qRT-PCR primer-*MLLT3*-R | GTGGTGGAGGTTCGTGATGT |
| qRT-PCR primer-*MYCT1*-F | CCATCAGCACTTCCCACAGT |
| qRT-PCR primer-*MYCT1*-R | TGAAAGGCCCACTCGAAGAC |
| qRT-PCR primer-*CD90*-F | GCAGAAGGTGACCAGCCTAA |
| qRT-PCR primer-*CD90*-R | TGCTTCTTTGTCTCACGGGT |
| qRT-PCR primer-*ANGPTL2*-F  qRT-PCR primer-*ANGPTL2*-R  qRT-PCR primer-*CD133*-F  qRT-PCR primer-*CD133*-R | GAGAACACCAACCGCCTCA  CCTTGCTTGTACGTCTCCCA  TGTGCGGGAACTCCTTTTCA  TGCCAATGGGTCCAGCTTTA |
